# LIFE-COURSE PERSPECTIVES ON MILITARY SERVICE EXPERIENCES, STRESS EXPOSURE, AND LATER- AND END-OF-LIFE OUTCOMES

**DOI:** 10.1093/geroni/igae098.0940

**Published:** 2024-12-31

**Authors:** Andrew London

**Affiliations:** Chair

## Abstract

Gerontological and life-course researchers continue to address gaps in knowledge related to the lived experiences and later-life outcomes of aging veterans. Aging veterans are similar to and distinct from aging non-veterans, and there is heterogeneity in the military and civilian experiences of veterans that is important for researchers to consider. This symposium features five presentations that expand existing knowledge about aging veterans. Spiro and colleagues focus on the long-term persistence of post-traumatic stress disorder (PTSD) and find it correlated with greater depression, anxiety, and disability, with worse health functioning and more mental health utilization. Kurth and colleagues examine stress exposure over a 20-year period and find a potential long-term advantage for aging combat veterans’ well-being related to reduced avoided arguments. Yorgason and colleagues study cognitive functioning and find that veteran status is associated with worse overall cognitive functioning, especially among veterans with PTSD. Ream and colleagues find that rural providers report their clients experience more unmet social needs, but also report positive factors associated with residence in rural areas. Additionally, they report that 80 percent of rural providers are interested in delivering a group-based intervention: Enhancing Social Functioning for Older Veterans with PTSD (ESVP). Berry-Stoelzle and colleagues focus on end-of-life and identify the level of associated support services, formal inclusion of families, group identity, difference in the perceived outcome, time since military service, and branch as factors influencing advance directive completion. Collectively, these papers underscore the enduring influence of military service experiences on the health and well-being of aging veterans. Aging Veterans: Effects of Military Service across the Life Course Interest Group Sponsored Symposium

